# Correction: Sustainable waste management and recycling of Zn–Al layered double hydroxide after adsorption of levofloxacin as a safe anti-inflammatory nanomaterial

**DOI:** 10.1039/d0ra90083d

**Published:** 2020-08-06

**Authors:** Samar M. Mahgoub, Mohamed R. Shehata, Fatma I. Abo El-Ela, Ahmed Farghali, Amal Zaher, Rehab K. Mahmoud

**Affiliations:** Department of Environmental Science and Industrial Development, Faculty of Postgraduate Studies for Advanced Sciences, Beni-Suef University 62511 Beni-Suef Egypt; Chemistry Department, Faculty of Science, Cairo University Giza Egypt; Department of Pharmacology, Faculty of Veterinary Medicine, Beni-Suef University Beni-Suef Egypt; Materials Science and Nanotechnology Department, Faculty of Postgraduate Studies for Advanced Sciences, Beni-Suef University Egypt; Department of Chemistry, Faculty of Science, Beni-Suef University 62511 Beni-Suef Egypt DR.Rehab.khaled@science.bsu.ed radwaraft@yahoo.com

## Abstract

Correction for ‘Sustainable waste management and recycling of Zn–Al layered double hydroxide after adsorption of levofloxacin as a safe anti-inflammatory nanomaterial’ by Samar M. Mahgoub *et al.*, *RSC Adv.*, 2020, **10**, 27633–27651. DOI: 10.1039/D0RA04898D.

The authors regret that, in the originally published version of this article, the name of the author Fatma I. Abo El-Ela was incorrectly displayed as Fatma L. Abo El-Ela. The correct author list is displayed above.

The Royal Society of Chemistry apologises for these errors and any consequent inconvenience to authors and readers.

## Supplementary Material

